# An Unstructured Supplementary Service Data–Based mHealth App Providing On-Demand Sexual Reproductive Health Information for Adolescents in Kibra, Kenya: Randomized Controlled Trial

**DOI:** 10.2196/31233

**Published:** 2022-04-15

**Authors:** Paul Macharia, Antoni Pérez-Navarro, Betsy Sambai, Irene Inwani, John Kinuthia, Ruth Nduati, Carme Carrion

**Affiliations:** 1 Universitat Oberta de Catalunya Barcelona Spain; 2 Kenyatta National Hospital Nairobi Kenya; 3 University of Nairobi Nairobi Kenya

**Keywords:** adolescents, sexual reproductive health, mobile phones, randomized controlled trial

## Abstract

**Background:**

Adolescents transitioning from childhood to adulthood need to be equipped with sexual reproductive health (SRH) knowledge, skills, attitudes, and values that empower them. Accessible, reliable, appropriate, and friendly information can be provided through mobile phone–based health interventions.

**Objective:**

This study aims to investigate the effectiveness and impact of an Unstructured Supplementary Service Data (USSD)–based app in increasing adolescents’ knowledge about contraceptives, gender-based stereotypes, sexually transmitted infections (STIs), abstinence, and perceived vulnerability, and helping adolescents make informed decisions about their SRH.

**Methods:**

A randomized controlled trial (RCT) methodology was applied to investigate the potential of a USSD-based app for providing on-demand SRH information. To be eligible, adolescents aged 15 to 19 years residing in Kibra, Kenya, had to have access to a phone and be available for the 3-month follow-up visit. Participants were randomly assigned to the intervention (n=146) and control (n=154) groups using sequentially numbered, opaque, sealed envelopes. The primary outcome was improved SRH knowledge. The secondary outcome was improved decision-making on SRH. The outcomes were measured using validated tools on adolescent SRH and user perceptions during the follow-up visit. A paired sample *t* test was used to compare the changes in knowledge scores in both groups. The control group did not receive any SRH information.

**Results:**

During the RCT, 54.9% (62/109) of adolescents used the USSD-based app at least once. The mean age by randomization group was 17.3 (SD 1.23) years for the control group and 17.3 (SD 1.12) years for the intervention group. There was a statistically significant difference in the total knowledge scores in the intervention group (mean 10.770, SD 2.012) compared with the control group (mean 10.170, SD 2.412) conditions (*t*_179_=2.197; *P*=.03). There was a significant difference in abstinence (*P*=.01) and contraceptive use (*P*=.06). Of the individuals who used the app, all participants felt the information received could improve decision-making regarding SRH. Information on STIs was of particular interest, with 27% (20/62) of the adolescents seeking information in this area, of whom 55% (11/20) were female. In relation to improved decision-making, 21.6% (29/134) of responses showed the adolescents were able to identify STIs and were likely to seek treatment; 51.7% (15/29) of these were female. Ease of use was the most important feature of the app for 28.3% (54/191) of the responses.

**Conclusions:**

Adolescents require accurate and up-to-date SRH information to guide their decision-making and improve health outcomes. As adolescents already use mobile phones in their day-to-day lives, apps provide an ideal platform for this information. A USSD-based app could be an appropriate tool for increasing SRH knowledge among adolescents in low-resource settings. Adolescents in the study valued the information provided because it helped them identify SRH topics on which they needed more information.

**Trial Registration:**

Pan African Clinical Trial Registry PACTR202204774993198; https://pactr.samrc.ac.za/TrialDisplay.aspx?TrialID=22623

## Introduction

### Background

The World Health Organization (WHO) has stated that universal access to quality sexual reproductive health (SRH) services is essential for sustainable development and global realization of health and human rights [1]. The United Nations has also made a commitment to ensure “universal access to sexual health and reproductive health-care services, including family planning, information and education” [2]. As SRH rights are fundamental to humanity’s well-being, the provision of evidence-based SRH interventions will secure lifelong positive impacts on health benefits and outcomes [3].

Adolescents transitioning from childhood to adulthood must be equipped with SRH knowledge, skills, attitudes, and values that empower them to develop successful sexual relationships. Several approaches, including comprehensive sexuality education and curriculum-based approaches, have been used to teach adolescents different aspects of sexuality [4]. Health interventions can be expanded to settings that adolescents engage in, beyond family and health care facilities [5]. Adolescents can be provided with high-impact, easily accessible, and reliable health information that is crucial for improving their reproductive health [6]. Content should be adolescent-friendly, appropriate to their SRH needs, swiftly provided, and not overwhelming [7].

In low- and middle-income countries, the exponential growth of mobile-based technologies has provided opportunities for the adoption of mobile health (mHealth) apps. The WHO identifies many mobile phone technologies that can be used to improve health outcomes in low- and middle-income countries, including SMS text messaging [8]. As research has shown, modes of information delivery and content must vary according to audience, appealing to different users in different ways [9]. In resource-limited settings, for instance, technology-based interventions have proven to be an effective way of providing health information [10].

Using evidence-based content to deliver adolescent SRH information on mobile phones has the potential to impact behaviors and improve health outcomes [11]. Several mobile phone–based interventions providing adolescent SRH services and their impact have been well-documented [12]. Research has shown that mHealth interventions have the potential to engage adolescents across sociodemographic settings and increase their knowledge and awareness [13]. Such interventions appeal to adolescents and, therefore, can mitigate the barriers to access associated with the delivery of adolescent SRH information at health care facilities [14].

Mobile phone–based health interventions are an increasingly feasible way to connect adolescents with SRH information and services in low-resource settings. Research has shown that interventions have been able to provide adolescents with knowledge that can lead to behavior change and improved health outcomes [15]. Mobile phone–based interventions can be tailored to each adolescent’s context and provide individualized and effective services [16]. To improve such interventions, it is important to document and review system interaction data to inform design and delivery improvements, thereby making mHealth apps more effective [17]. There are often concerns about privacy when using mobile phone apps, which must be considered during the app development process [15]. Unstructured Supplementary Service Data (USSD)-based mobile phone technology has been found to be a user-friendly, convenient, and confidential method for adolescent users to access SRH information [18,19].

### Objective

This study investigates the potential of a USSD-based app for providing on-demand SRH information to adolescents in the resource-limited setting of Kibra, Nairobi County, Kenya. The aim of this study is to determine the effectiveness and impact of a USSD-based mobile phone app in (1) increasing adolescents’ knowledge about contraceptives, gender-based stereotypes, sexually transmitted infections (STIs), abstinence, and perceived vulnerability and (2) helping adolescents make informed decisions about their SRH.

## Methods

### Ethics Approval

The study protocol was reviewed and approved by the Kenyatta National Hospital University of Nairobi Ethics Review Committee in March 2019 (reference number P707/10/2018).

### Intervention Design

The intervention design was based on the health belief model, a behavior change framework intended to increase knowledge that can inform actions to reduce health risks [20]. Through a randomized controlled trial (RCT), a USSD-based app was evaluated on its ability to influence adolescents’ knowledge, attitudes, and practices related to SRH health awareness. Content provided in the app was based on validated adolescent sexual health information (Multimedia Appendix 1) created through Avert’s Young Voices, a project that developed materials and content on adolescent sexual health through a co-creation process with adolescents from South Africa, Lesotho, Zambia, Zimbabwe, and Malawi [21].

### Sample Size

The study enrolled 300 adolescents: 146 (48.7%) randomized to the intervention group and 154 (51.3%) to the control group. It is estimated that around 8% of adolescents aged 15 to 19 years in Kenya access SRH information [22]. A minimum sample size of 226 adolescents was required to attain a 95% CI. Thus, the sample size of 300 adolescents had 74 more participants than the minimum sample size. The additional participants ensured that the sample strength would be maintained, even with loss at follow-up.

The study used sequentially numbered, opaque sealed envelopes—an affordable and effective method for randomizing participants [23]. Having passed screening for eligibility, the adolescents picked a sealed envelope from a box. Each envelope contained a randomization group and an assigned participant ID number. A randomization list was generated using a web-based tool [24].

### Recruitment

The study population consisted of adolescents aged 15 to 19 years residing in the Kibra suburb of Nairobi City County, Kenya. Kibra consists of 12 villages, with both formal and informal settlements. The informal settlements house approximately 2.5 million residents. Participants were mobilized from 12 villages; community mobilizers approached potential participants at social halls, sports events, and other social activities that attracted adolescents aged 15 to 19 years. The study procedures were explained individually or to small groups of 3 to 5 adolescents using a study recruitment script (Multimedia Appendix 2). Adolescents interested in the study were referred to the study venue.

**Figure 1:**
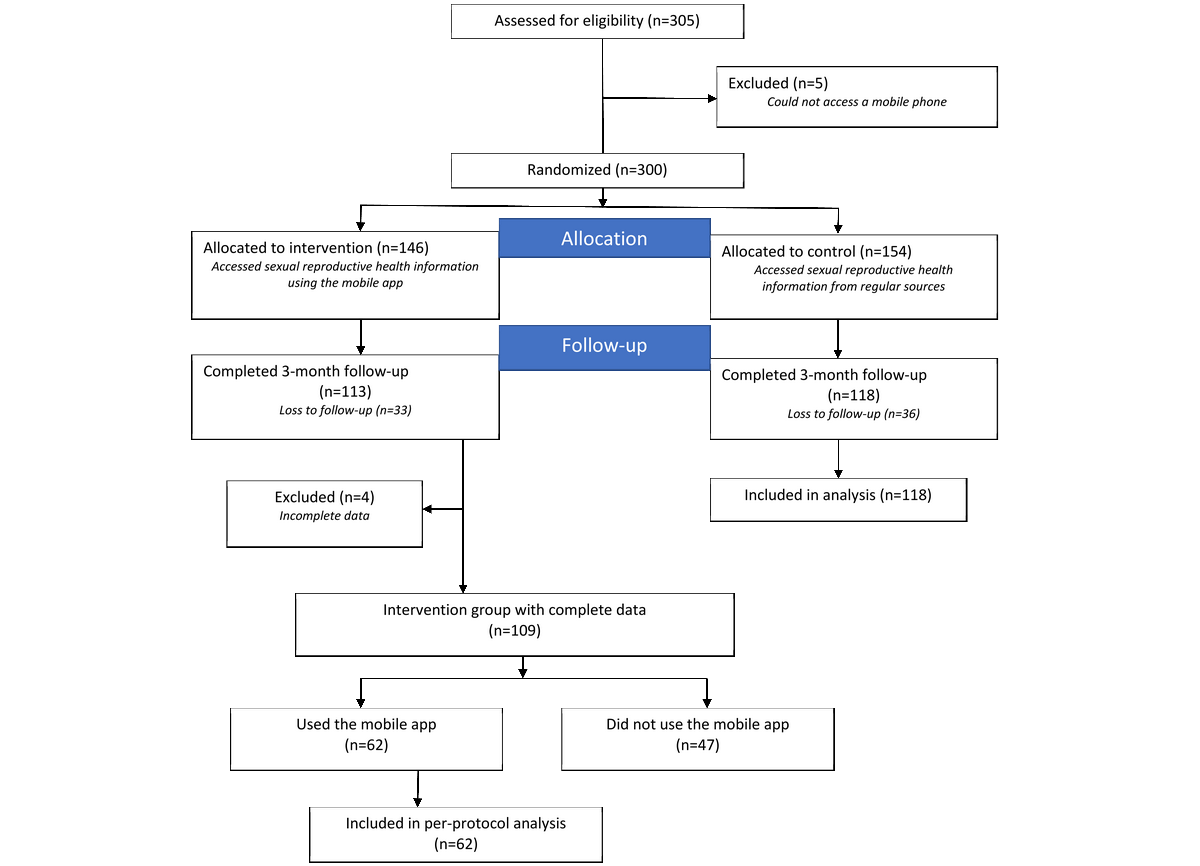
Study participant flowchart.

Efforts were made to distribute enrollment across all villages, as there are intervillage ethnic differences. Enrollment numbers were monitored by village and randomization groups during enrollment to ensure equitable distribution, providing an improved representation of adolescent SRH needs and awareness across the area. Ethnicity data were not collected because of the stigma associated with issues or discussions on ethnicity in the study site setting. We distributed enrollment across all the villages, ensuring a true representation of Kibra. In 2 cases, the enrollment team moved the study site to a social hall near a particular village to make it easier for local adolescents to participate. Figure 1 shows the enrollment and follow-up processes.

### Inclusion and Exclusion Criteria

To participate in the study, adolescents should be aged between 15 and 19 years, live in Kibra, and be able to access a mobile phone. Participants aged 15 to 17 years signed an assent form after assenting to the study procedures; those aged ≥18 years were required to sign a consent form.

Adolescents aged <18 years should be accompanied by a parent or guardian, should get the permission from the parent or guardian, and should provide their assent. As the study presented minimal risk, the study team requested a waiver of parental permission for adolescents aged <18 years who were unaccompanied. In this setting, there are cultural challenges related to discussions with parents regarding adolescent SRH. Parents or guardians in Kibra, as in many settings, may not be involved in or fully aware of their adolescents’ SRH information needs. If the study opted to secure parental permission for adolescents participating in the study, this may have required disclosure of the participants’ SRH information needs, potentially leading to an elevated risk of harm or prevention of participation.

### Intervention Implementation and Data Collection

At the study venue, all potential participants were provided with further details of the study, eligibility criteria, and study procedures. On the basis of their age, an approved informed consent or assent form was provided in either English or Swahili. Potential participants were given time to ask questions, and after these were addressed, the study staff verified whether the potential participants were still interested. After the study procedures were explained in detail, participants signed a consent or assent form. Study staff then signed and dated the consent or assent forms, and participants were provided with a copy, if desired. For adolescents in the control group, no SRH information was provided. It was assumed that these adolescents would get information from their regular sources, including their parents, peers, or seminars held by nongovernmental organizations in Kibra.

The *Evaluation of Knowledge of SRH Information* (Multimedia Appendix 3) and *Use and Perception of the Mobile Phone App* (Multimedia Appendix 4) questionnaires were administered to adolescents by study staff. Open Data Kit was used to administer the 2 questionnaires. This mobile app enables a survey to be administered through a smartphone, question by question, in an easy-to-use, user-friendly interface. The *Evaluation of Knowledge of SRH Information* questionnaire was administered at enrollment and follow-up visits. The *Use and Perception of the Mobile Phone App* questionnaire was only administered to adolescents in the intervention group who had used the app at least once.

**Figure 2:**
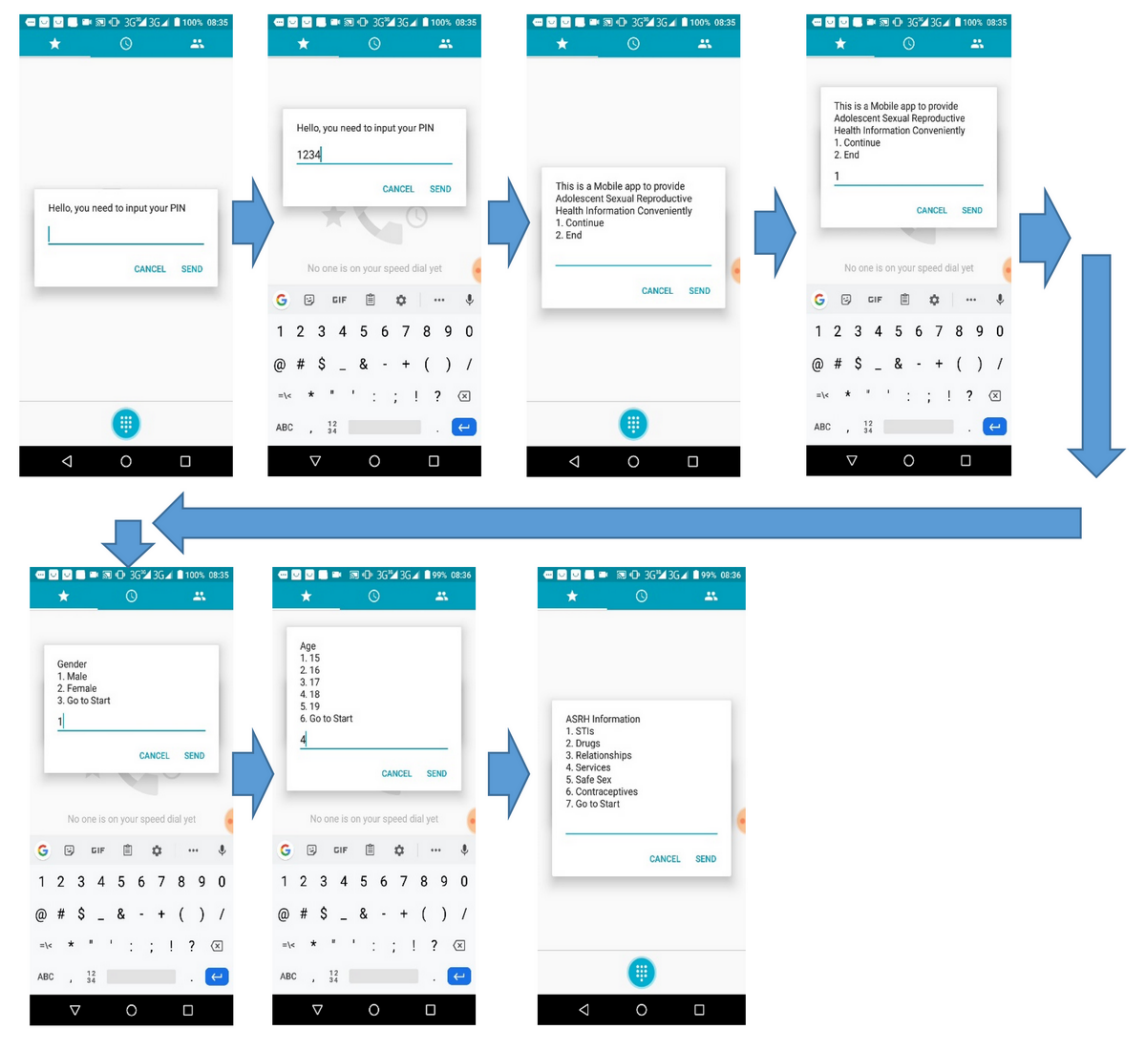
Connecting to the Unstructured Supplementary Service Data (USSD) app. STI: sexually transmitted infection.

For the follow-up visit, community mobilizers called each participant, requesting them to visit the study site. Follow-up interviews were scheduled based on adolescents’ availability. A USSD app offering validated SRH information accessible on both feature phones and smartphones was availed to the adolescents in the intervention group. To visualize the USSD app, Figure 2 shows the layout of the app and its interactive screens. A user-centered design approach was used in the design and development of the USSD app [18].

### Sexual Reproductive Health Knowledge Score

To evaluate intervention outcomes, the *Evaluation of Knowledge of SRH Information* questionnaire (Multimedia Appendix 3) based on Monitoring and Evaluation to Assess and Use Results evaluation indicators on adolescent SRH was administered during the 3-month follow-up visit [25]. The knowledge scores calculated from these questionnaires were used to evaluate awareness.

The questions required yes or no responses. Each correct response earned one point and the wrong answer scored zero. Questions were posed to adolescents in both the intervention and control groups at enrollment and follow-up visits. The knowledge score data were analyzed as an aggregate score and by each subsection of the *Evaluation of Knowledge of SRH Information* questionnaire (Multimedia Appendix 3). Only the questions in section 2 of the questionnaire were used to evaluate knowledge scores. All estimates were adjusted for age, sex, access to phones, and level of education.

### Use and Perceptions of the Mobile Phone App

The *Use and Perception of the Mobile Phone App* questionnaire (Multimedia Appendix 4) was administered to participants who had used the app at least once during the 3-month period. This questionnaire is based on WHO-validated instruments intended to be used by investigators studying SRH among adolescents [26] and assesses potentially improved decision-making. Knowledge of contraceptive use, STIs, and abstinence was also assessed. This study paid particular attention to detailed descriptions of recent encounters to determine the intervention’s impact on improved awareness of SRH information.

### Statistical Analysis

#### Overview

A paired sample *t* test was conducted to compare the knowledge score data. The *t* test attempted to show if there were differences in knowledge scores between the intervention and control groups at the 3-month follow-up visit. We also attempted to assess changes in attitude toward contraceptives, gender role stereotypes, abstinence, and perceived vulnerability. The data were analyzed using R software (R Core Team).

For the *Use and Perception of the Mobile Phone App* questionnaire data, descriptive statistics were used to assess adolescents’ knowledge, awareness, and potentially improved decision-making in relation to SRH. A chi-square test was used to assess any differences by age group, with a *P* value <.05 regarded as significant. Data analysis was performed using the R software (version 3.6.2).

#### Univariate Analyses

Exploratory data analysis techniques were performed to reveal the distribution structure of the outcome variables and identify outliers or unusually entered values. Statistical analyses were performed using descriptive statistics for continuous (mean and SD) and categorical (frequency and proportion) variables. These tests were performed on each participant’s demographics and the *Use and Perception of the Mobile Phone App* data.

#### Bivariate Analysis

The distribution of the background characteristics of the study groups was compared. To establish baseline equivalence between the intervention and control groups, 2 analytical tests were used. The distribution of categorical variables (proportions) was compared using Pearson chi-square test, whereas the distribution of continuous variables (mean and SD) was compared using an independent *t* test. Bivariate analysis was also performed on participant demographics, and the use and perception of mobile phone app data. A *P* value <.05 was regarded as significant.

#### Analysis of the Effect of the Intervention

Longitudinal continuous outcome scores were analyzed across time points (baseline and end line) to understand the effect of variations in outcome scores. The 2-tailed paired sample *t* test would compare the means of the intervention and control groups. The continuous outcomes were normally distributed. The threshold for statistical significance for all analyses was set at *P*<.05. This analysis was performed on the knowledge score data.

## Results

### Overview

In October 2019, 305 adolescents from 12 villages in Kibra were mobilized and screened for eligibility. Owing to lack of access to a mobile phone, 5 adolescents were excluded from the study. Study participants were then randomized to the intervention (154/300, 51.3%) and control (146/300, 48.7%) groups. From late December 2019, 77% (231/300) of the adolescents were successfully followed up—74.7% (109/300) from the intervention group and 76.6% (118/300) from the control group. As the app was not used at least once, 47 participants, together with another 4 participants with incomplete data in the intervention group, were excluded from the final analysis. Figure 1 shows the enrollment and follow-up stages of the study. The data were analyzed as per the per-protocol analysis.

### Background Characteristics of the Study Participants

The distribution of the study participants according to the selected background characteristics indicated a desired comparable result at baseline as shown in Table 1. The mean ages of the participants in the control group (17.29, SD 1.23 years) and the intervention group (17.27, SD 1.12 years) were statistically comparable (*P*=.94). There were no significant differences in participant demographics. However, there was a statistically significant difference in the distribution of sex (*P*=.03) by the study enrollment group.

**Table 1:** Characteristics of the study participants (N=300).

Variables		Total (n=180), n (%)	Intervention (n=62), n (%)	Control (n=118), n (%)	*P* value	
**Sex**						.03
	Male	67 (37.2)	30 (48.4)	37 (31.4)		
	Female	113 (62.8)	32(51.6)	81 (68.6)		
**Phone ownership**						.64
	Adolescent	81 (45)	30 (48.4)	51 (43.2)		
	Parent or guardian	90 (50)	30 (48.4)	60 (50.8)		
	Other	9 (5)	2 (3.2)	7 (5.9)		
**Highest level of education**						.97
	Primary	23 (12.8)	8 (12.9)	15 (12.7)		
	Secondary and above	157 (87.2)	54 (87.1)	103 (87.3)		

### SRH Knowledge Score

Participants’ responses were analyzed by attitude toward contraceptives, gender role stereotypes, abstinence, and perceived vulnerability to negative SRH outcomes. Knowledge scores were also analyzed as aggregated data. A paired sample *t* test analysis of the relationship between the knowledge score and the use of the mobile app was performed using R software.

Table 2 presents an analysis of the effect of the intervention on specific indicator scores. The difference in the mean scores between those enrolled in the intervention group compared with those in the control group showed statistical significance in the total knowledge scores. The overall mean change in total scores in the intervention group was 0.5 (*P*=.02) compared with the control group 0.246 (*P*=.24). The *P* value between the 2 groups on the total knowledge scores was .03, which was statistically significant, indicating that the mobile app had an impact on the adolescents’ SRH knowledge scores. In the intervention group, the intervention had a statistically significant effect on contraceptive scores (0.355; *P*=.02). The intervention also showed a trend toward statistical significance in abstinence knowledge scores (0.129, *P*=.09).

**Table 2:** Effects of intervention on overall and specific knowledge scores.

Outcome (knowledge score)	Intervention						Control						Between group, *P* value
	Baseline, mean (SD); 95% CI	End line, mean (SD); 95% CI	Difference in scores, mean (SD); 95% CI	Effect sizes	Within group, *P* value	Baseline, mean (SD); 95% CI		End line, mean (SD); 95% CI	Difference in score, mean (SD); 95% CI	Effect sizes	Within group, *P* value		
Contraceptives	3.613 (1.107); 3 to 4	3.968 (0.887); 4 to 5	0.355 (1.147); 0.064 to 0.646	0.309	.02	3.602 (1.039); 3 to 4		3.678 (1.183); 3 to 5	0.076 (1.235); −0.148 to 0.301	0.062	.5	.06	
Vulnerability	2.000 (0.768); 1.25 to 3	2.032 (0.768); 2 to 3	0.0323 (0.829); –0.178 to 0.243	0.038	.76	1.856 (0.860); 1 to 2.75		1.941 (0.798); 1 to 2.75	0.085 (0.939); −0.086 to 0.256	0.090	.33	.32	
Gender stereotype	3.097 (0.987); 3 to 4	3.081 (1.060); 3 to 4	−0.016 (0.757); −0.208 to 0.176	0.021	.87	2.890 (1.160); 2 to 4		2.881 (1.126); 2 to 4	−0.008 (1.121); −0.213 to 0.196	0.008	.94	.88	
Abstinence	1.565 (0.532); 1 to 2	1.694 (0.465); 1 to 2	0.129 (0.586); −0.020 to 0.278	0.220	.09	1.576 (0.576); 1 to 2		1.669 (0.539); 1 to 2	0.129 (0.569); −0.011 to 0.197	0.163	.08	.01	
Total knowledge score	10.270 (2.050); 9 to 12	10.770 (2.012); 10 to 12	0.5 (1.576); 0.099 to 0.900	0.317	.02	9.924 (2.227); 8.25 to 12		10.170 (2.412); 9 to 12	0.246 (2.242); −0.163 to 0.654	0.109	.24	.03	

### Use and Perceptions of the Mobile Phone App

The use and perceptions questionnaires were used to measure the perceived usefulness of the app. We also aimed to evaluate how the knowledge adolescents received from the app influenced their SRH decision-making. Tables 3 and 4 show the descriptive statistics of our evaluation. The tables show the responses from each adolescent who had used the mobile app at least once in 3 months. The questions addressed topics of interest, the perceived usefulness of information, and the mobile app features the users appreciated. The information in Table 3 is stratified by age—adolescents aged <18 years and those ≥18 years. Table 4 is stratified by gender.

Information about STIs was of great interest to the participants, with 26.7% (20/75) of the responses by users seeking information on this subject the last time they used the app. Adolescent girl participants had a higher interest in STIs, with 55% (11/20) accessing this information. Most participants (56/62, 90.8%) found the information provided in the app to have adequately answered their questions or met their SRH information needs. All the 62 adolescents who used the app felt that the information they received could improve their decision-making on issues relating to SRH. This outcome was similar when data were stratified by age and gender.

The participants reported gaining knowledge from the app on several SRH issues in their responses, including abstinence (53/125, 42.4%), STIs (30/125, 24%), and condom use (22/125, 17.6%). Although only 9.7% (12/125) of the participant’s responses showed increased knowledge of contraceptives, 75% (9/12) of these were female, showing a trend toward significance (*P*=.08).

On improved decision-making, 38.1% (51/134) of the adolescent participant’s responses show they were able to abstain from sex. Of these responses, 54.9% (28/51) were aged between 15 and 17 years and 52.9% (27/51) were male. The knowledge obtained may have also prompted 26.9% (36/134) of the responses to show use a condom by the adolescent participants during a sexual encounter. Although sex is illegal for ages under 18 years in Kenya, 50% of those who reported deciding to use a condom were aged ≤17 years. Of the participants who used a condom, 52.8% (19/36) were male. Adolescent participants were also able to identify STIs, with 21.6% (29/134) responses reporting that app information guided their decision to seek treatment after identifying an STI; 51.7% (15/29) of these responses were from female participants.

Ease of use was the most important feature of the app for 28.3% (54/191) of the participants’ responses, followed by confidentiality at 26.7% (51/191) and high-quality information at 23.6% (45/191), with 60% (27/45) of the latter being from responses by female participants.

**Table 3:** Use and perception of the mobile app stratified by age groups (62 participants).

Variable			All, n (%)		Age <18 years, n (%)	Age ≥18 years, n (%)		*P* value	
**What information did you require when you last used the mobile app?**									
	STIs^a^	20 (26.7)		8 (40)		12 (60)	.37		
	Drugs	18 (24)		12 (66.7)		6 (33.3)	.16		
	Relationship	17 (22.7)		9 (52.9)		8 (47.1)	.81		
	Sex	12 (16)		4 (33.3)		8 (66.7)	.25		
	Contraceptives	6 (8)		3 (50)		3 (50)	>.99		
	Pregnancy	2 (2.7)		0 (0)		2 (100)	.16		
**What knowledge about SRH^b^** **issues have you gained?**									
	Abstinence	53 (42.4)		28 (52.8)		25 (47.2)	.68		
	STIs	30 (24)		14 (46.7)		16 (53.3)	.72		
	Condom use	22 (17.6)		12 (54.5)		10 (45.5)	.67		
	Contraceptives	12 (9.6)		6 (50)		6 (50)	>.99		
	Drugs	8 (6.4)		5 (62.5)		3 (37.5)	.48		
**What decision-making was informed by the information you accessed on the mobile app?**									
	Abstinence	51 (38.1)		28 (54.9)		23 (45.1)	.48		
	Condom use	36 (26.9)		18 (50)		18 (50)	>.99		
	STIs	29 (21.6)		11 (37.9)		18 (62.1)	.19		
	Contraceptives	9 (6.7)		6 (66.7)		3 (33.3)	.32		
	Drugs	9 (6.7)		6 (66.7)		3 (33.3)	.32		
**Were the questions you had on SRH answered adequately?**									
	Yes	56 (90.3)		30 (53.6)		26 (46.4)	>.99		
	No	6 (9.7)		3 (50)		3 (50)			
**Did the information you receive inform better decision-making on SRH matters?**									
	Yes	62 (100)		33 (53.2)		29 (46.8)	.62		
	No	0 (0)		0 (0)		0 (0)			
**What are the most important features of the mobile phone app?**									
	Ease of use	54 (28.3)		28 (51.9)		26 (48.1)	.79		
	Confidentiality	51 (26.7)		26 (51)		25 (49)	.89		
	Quality of information	45 (23.6)		24 (53.3)		21 (46.7)	.65		
	Immediate feedback	41 (21.5)		20 (48.8)		21 (51.2)	.88		

^a^STI: sexually transmitted infection.

^b^SRH: sexual reproductive health.

**Table 4:** Use and perception of the mobile app stratified by gender (62 participants).

Variable			All, n (%)		Male, n (%)			Female, n (%)			*P* value	
**What information did you require when you last used the mobile app?**												
	STIs^a^	20 (27)		9 (45)			11 (55)			.65		
	Drugs	18 (24.3)		10 (55.6)			8 (44.4)			.64		
	Relationships	16 (21.6)		8 (47.1)			9 (52.9)			.81		
	Sex	12 (16.2)		5 (41.7)			7 (58.3)			.56		
	Contraceptives	6 (8.1)		1 (16.7)			5 (83.3)			.10		
	Pregnancy	2 (2.7)		1 (50)			1 (50)			>.99		
**What knowledge about sexual reproductive health matters have you gained?**												
	Abstinence	53 (42.7)				26 (49.1)			27 (50.9)			.89
	STIs	30 (24.2)				13 (43.3)			17 (56.7)			.47
	Condom use	22 (17.7)				13 (50.1)			9 (40.9)			.39
	Contraceptives	12 (9.7)				3 (25)			9 (75)			.08
	Drugs	7 (5.6)				2 (25)			6 (75)			.16
**What better decision-making was informed by the information you accessed on the mobile app?**												
	Abstinence	51 (38.1)				27 (52.9)			24 (48.1)			.67
	Condom use	36 (26.9)				19 (52.8)			17 (47.2)			.74
	STIs	29 (21.6)				14 (48.3)			15 (51.7)			.85
	Contraceptives	9 (6.7)				4 (44.4)			5 (55.6)			.74
	Drugs	9 (6.7)				3 (33.3)			6 (66.7)			.32
**Were the questions you had on SRH^b^ answered adequately?**												
	Yes	56 (90.3)		26 (46.4)			30 (53.6)			.61		
	No	6 (9.7)		4 (33.3)			2 (66.7)					
**Did the information you receive inform better decision-making on SRH matters?**												
	Yes	62 (100)				30 (48.4)			32 (51.6)			.80
	No	0 (0)				0			0			
**What are the most important features of the mobile phone app?**												
	Easy to use	54 (28.3)				26 (48.1)	28 (51.9)			.79		
	Confidentiality	51 (26.7)				24 (47.1)	27 (52.9)			.67		
	Quality of information	45 (23.6)				18 (40)	27 (60)			.18		
	Immediate feedback	41 (21.5)				19 (46.3)	22 (53.7)			.64		

^a^STI: sexually transmitted infection.

^b^SRH: sexual reproductive health.

## Discussion

### Principal Findings

This study explored the use of a USSD-based mobile phone intervention to deliver on-demand adolescent SRH information in an RCT. We studied the effectiveness and impact of a USSD-based mobile phone app on increasing adolescents’ knowledge of contraceptives, gender-based stereotypes, STIs, abstinence, and perceived vulnerability. We also evaluated the USSD-based ability of the mobile phone app to help adolescents make informed decisions regarding their SRH. Our results show improved awareness of SRH information and improved knowledge about contraceptives and abstinence. Increased awareness has enabled more adolescents to abstain from sex, improve condom use, and identify STIs. Confidentiality when accessing SRH information was of particular importance to the participants.

Adolescents’ needs for information on contraceptives is unmet in most resource-limited settings; adolescents are unable to secure information on available contraceptive options or discover where they can access this information [27]. In our study, adolescents using the app improved their knowledge of contraceptives, with a trend toward statistical significant (*P*=.06). Our findings are promising, and mobile phone apps could help increase awareness on and knowledge of contraceptives among adolescents. The provision of information on contraceptives to adolescents is complex because of cultural, religious, and political setbacks. Innovative approaches are needed to meet adolescents’ information needs. The study outcomes also show the need to make information about contraceptives accessible to adolescents in a culturally and age-appropriate manner [28,29].

When accessing SRH information and services, adolescents want their confidentiality to be respected and upheld. Fear of being *judged* and the possibility of negative attitudes from health care providers can prevent adolescents from accessing these important services [30]. During follow-up visits, of the 62 adolescent participants who had used the mobile app, 51 (82.3%) indicated that confidentiality was one of the most important features of the app. Adolescent users can access any SRH information in a user-friendly manner. Research has shown that adolescents value confidentiality when accessing SRH information and are more willing to seek SRH care and interventions when their confidentiality is assured [31].

mHealth apps have shown great potential for engaging with and increasing SRH information access for adolescents from different age groups and social demographics [32,33]. In one study, text messages improved SRH outcomes by reducing pregnancy rates [34]. The aforementioned studies demonstrate the great potential of mHealth apps in improving and increasing adolescents’ knowledge of SRH. Our study findings show that adolescents require high-quality SRH information provided in an easy-to-use, confidential manner with immediate feedback. The USSD technology enables an interactive user-driven mobile app to provide information based on a user’s inputs. This USSD technology is low cost, works on both feature phones and smartphones, and can be provided free of charge.

### Limitations

During the study, 47 adolescents were unable to use the mobile app, mainly because of a lack of access to mobile phones. This may explain why there appeared to have been a minimal change in adolescent users’ knowledge scores. Access to mobile phones in most resource-limited settings is associated with the household economic status. In addition, access to a phone was self-reported. Several adolescents hoped to be provided with a phone by their parents, caregivers, or older siblings. Adolescent participants in the intervention group who were unable to use the app reported that either their parents traveled or the mobile phone they hoped to use stopped functioning. Some studies have opted to provide adolescent participants with mobile phones to ensure that participants in the intervention group accessed the mobile apps. This approach has increased the cost of the study, and other researchers have viewed providing mobile phones as an inducement. In resource-limited settings such as Kenya, access to the internet is limited and web-based apps may not be an option in this setting. However, internet cybercafes are available in many places. Providing internet payment vouchers to adolescents to access the internet and a customized web-based study app could be explored. The results of our study may not be generalizable across Kibra.

### Conclusions

Adolescents require accurate and up-to-date SRH information to guide their decision-making and improve health outcomes. As they already use mobile phones in their day-to-day lives, mobile phone apps provide an ideal platform. Considerable promise has been demonstrated by studies using mobile apps to improve adolescents’ access to SRH information. Scaled-up research on mHealth apps providing SRH information is required to better evaluate their impact on SRH outcomes.

## Appendix

Multimedia Appendix 1 Unstructured Supplementary Service Data app content.

Multimedia Appendix 2 Adolescent recruitment script page of the approved consent form in English.

Multimedia Appendix 3 Evaluation of knowledge of sexual reproductive health (SRH) information.

Multimedia Appendix 4 Use and perceptions of the mobile phone app.

Multimedia Appendix 5 CONSORT-EHEALTH (V 1.6.1) checklist.

## Abbreviations

mHealth: mobile health
RCT: randomized controlled trial
SRH: sexual reproductive health
STI: sexually transmitted infection
USSD: Unstructured Supplementary Service Data
WHO: World Health Organization
