# Supplementary material for: An Unstructured Supplementary Service Data–Based mHealth App Providing On-Demand Sexual Reproductive Health Information for Adolescents in Kibra, Kenya: Randomized Controlled Trial
Source: JMIR Mhealth Uhealth. 2022 Apr 15;10(4):e31233. doi: 10.2196/31233 (PMC9055479; doi:10.2196/31233)
Supplement: Multimedia Appendix 1 [file mhealth_v10i4e31233_app1.pdf]

Appendix 1: Unstructured Supplementary Service Data App Content

No.	Question	Response
1	Gender	1. Male<br>2. Female
2	Age	1. 15 (go to 3b)<br>2. 16 (go to 3b)<br>3. 17 (go to 3b)<br>4. 18 (go to 3a)<br>5. 19 (go to 3a)
3	**ASRH Information**	
3a	List of options (Age 18 and above)	1. STIs Information (go to 4)<br>2. Contraceptives (go to 5)<br>3. Safe Sex (go to 13)<br>4. Abstain from Drugs (go to 3c)<br>5. Sexual Relationships (go to 6)<br>6. Adolescent friendly services (go to 18)
3b	List of options (Age 15–17)	1. STIs Information (go to 4)<br>2. Abstain from Sex (go to 13)<br>3. Abstain from Drugs (go to 3c)<br>4. Sexual relationship (go to 6)<br>5. Adolescent friendly services (go to 18)
3c	Abstain from Drugs	1. Drug use screening<br>2. Drugs and sex
4	STIs Information	3. STI Symptoms (go to 7)<br>4. Talking about HIV (go to 10)
5	Contraceptives	1. Options (go to 11)<br>2. Sex with no condom (go to 12)
6	Sexual relationships	1. Dating someone much older (go to 14)<br>2. Signs of unhealthy relationships (go to 15)
**STIs Information**		
7	**Symptomatic STI Screening** https://web.uri.edu/antimicrobial-stewardship/files/Sexually-Transmitted-Infections.pdf	1. Female (go to 8)<br>2. Male (go to 9)
8	**Symptomatic STI Screening** Females	
8a	Vaginal itching	1. Yes<br>2. No
8b	Vaginal discharge	1. Yes<br>2. No
8c	Painful urination	1. Yes<br>2. No
8d	Increased urinary urgency	1. Yes<br>2. No
8e	Pelvic pain	1. Yes<br>2. No
8f	Pain with sexual intercourse	1. Yes<br>2. No
8g	Vaginal bleeding	1. Yes<br>2. No
8h	Genital blisters	1. Yes<br>2. No
8i	Genital ulcer	1. Yes<br>2. No

8j	You need further assessment (If “Yes” in any of 8a – 8i go to 18)	
9	**Symptomatic STI Screening** Males	
9a	Penile discharge	1. Yes<br>2. No
9b	Painful urination	1. Yes<br>2. No
9c	Increased urgency	1. Yes<br>2. No
9d	Pelvic pain	1. Yes<br>2. No
9e	Swollen/tender testicles	1. Yes<br>2. No
9f	Pain with sexual intercourse	1. Yes<br>2. No
9g	Genital blisters	1. Yes<br>2. No
9h	Genital ulcer	1. Yes<br>2. No
9i	You need further assessment (If “Yes” in any of 9a – 9h go to 18)	1. Continue
**10. Talking about HIV with a partner** https://www.avert.org/hubs/young-voices-africa/talking-about-hiv		
10a	You don’t have to apologize because you are living with HIV.	1. Continue
10b	Have some information on hand to share with them.	1. Continue
10c	Think of the best time to tell them, not when in a rush or stressed.	1. Continue
10d	Find a place you are less likely to be interrupted.	1. Continue
10e	Whatever your partner’s first reaction, it could change over time.	1. Continue
10f	It’s important your partner gets tested. Practice safer sex in the future.	1. Continue
10g	Fear and stigma could stir up very strong emotions.	1. Continue
10h	Your status may make some people afraid or judgmental.	1. Continue<br>2. Main
**Contraceptives** https://www.avert.org/hubs/young-voices-africa/options-for-contraception		
11	**Options for contraception**	
11a	Many contraceptives only prevent pregnancies.	1. Continue
11b	How long lasting you want it to be; able to remember to take it.	1. Continue
11c	A good idea to talk about the options with partner.	1. Continue
11d	**Condoms** prevent both pregnancy and STIs.	1. Continue
11e	The **contraceptive pill** taken by women, prevents pregnancy but no protection from STIs.	1. Continue
11f	An **implant** for women prevents pregnancy. Lasts up to four years, no protection from STIs.	1. Continue

11g	**Injections** for women, prevent pregnancy. No protection from STIs.	1. Continue<br>2. Main
12	**Sex with no condom** https://www.avert.org/hubs/young-voices-africa/sex-without-a-condom	1. Continue
12a	Condom use, is one of the only protections from STIs and pregnancy.	1. Continue
12b	Talking about it beforehand, both clear on what you want.	1. Continue
12c	Partner pressurizing for sex without a condom is a sign they aren't taking care of themselves and you.	1. Continue
12d	Remember that you should never use a condom more than once.	1. Continue
12e	Neither should you double up, this creates friction, condoms could break.	1. Continue
12f	Condoms usually come pre-lubricated, but extra lubrication reduces STI risk.	1. Continue
12g	If using latex condoms, make sure you use a water-based lube.	1. Continue<br>2. Main
**13. Safe/Abstain from sex** https://www.avert.org/sex-stis/consent		
13a	You can say no at any time.	1. Continue
13b	Being in a relationship does not give your partner the right to do anything they want to you.	1. Continue
13c	You can change your mind about sex at any time.	1. Continue
13d	The legal age to have sex in Kenya is 18.	1. Continue
13e	Sexual contact without consent is wrong whatever the age of people involved.	1. Continue
13f	Difficult to say no in the heat of the moment – let partner know beforehand about your wishes.	1. Continue<br>2. Main
**Sexual Relationships**		
14	**Dating someone much older than you.** https://www.avert.org/hubs/young-voices-africa/dating-older-people	1. Continue
14a	The person will expect, or demand, things in return.	1. Continue
14b	The person is likely to want to be in control.	1. Continue
14c	The person could be living with HIV.	1. Continue
14d	The person is likely dating/having sex with other people.	1. Continue
14e	The person's expectations could be very different to yours.	1. Continue
14f	If some of your friends are dating older persons, still decide what's right for you.	1. Continue<br>2. Main

15	**Signs of unhealthy relationships** https://www.avert.org/hubs/young-voices-africa/unhealthy-relationships	
15a	Partner attempts to exercise control and power over you.	1. Continue
15b	Pressures you to send intimate photo messages that you don't want to.	1. Continue
15c	You don't feel able or willing to communicate with your partner.	1. Continue
15d	Your partner is being repeatedly dishonest.	1. Continue
15e	They call you names, constantly question and criticize your choices and decisions.	1. Continue
15f	You don't feel you have any control over whether to use contraceptives.	1. Continue
15g	You don't feel able to go out and see your friends without the partner being angry or sad or jealous.	1. Continue
15h	You are made to feel guilty for the choices that you make.	1. Continue<br>2. Main
**Abstain from Drugs**		
16	Adolescent Screening tool for drug use disorders https://crafft.org/	
**Part A**		
16a	During the PAST 12 MONTHS, did you: Drink any alcohol?	1. Yes<br>2. No
16b	During the PAST 12 MONTHS, did you: Smoke any marijuana or hashish?	1. Yes<br>2. No
16c	During the PAST 12 MONTHS, did you: Use anything else to get high?	1. Yes<br>2. No
If "Yes" in any of 16a – 16c go to Part B		
**Part B**		
16d	Have you ever ridden in a CAR driven by someone using alcohol or drugs?	1. Yes<br>2. No
16e	Do you ever use alcohol or drugs to RELAX, feel better about yourself, or fit in?	1. Yes<br>2. No
16f	Do you ever use alcohol or drugs while you are by yourself, or ALONE?	1. Yes<br>2. No
16g	Do you ever FORGET things you did while using alcohol or drugs?	1. Yes<br>2. No
16h	Do your FAMILY or FRIENDS ever tell you that you should cut down on your drinking or drug use?	1. Yes<br>2. No
16i	Have you ever gotten into TROUBLE while using alcohol or drugs?	1. Yes<br>2. No

16j	You need additional assessment.<br>(If "Yes" for two or more question 16a -16i)<br>(Go to 18e)	1. Continue
17	Risks of mixing drugs and sex	1. Continue
17a	Forget to use a condom	1. Continue
17b	Not be able to consent for sex	1. Continue
17c	Unaware of someone spiking your drink	1. Continue
17d	Engage in more risky sexual activities	1. Continue<br>2. Main
**Information on youth friendly services**		
18	The following are adolescent-friendly services in your neighborhood	1. Facility A (go to 18a)<br>2. Facility B (go to 18b)<br>3. Facility C (go to 18c)<br>4. Facility D (go to 18d)
18a	Contact details of facility A	
18b	Contact details of facility B	
18c	Contact details of facility C	
18d	Contact details of facility D	
18e	Contact details of facility E<br>(Drug use screening and referral)	
