# Supplementary material for: An Unstructured Supplementary Service Data–Based mHealth App Providing On-Demand Sexual Reproductive Health Information for Adolescents in Kibra, Kenya: Randomized Controlled Trial
Source: JMIR Mhealth Uhealth. 2022 Apr 15;10(4):e31233. doi: 10.2196/31233 (PMC9055479; doi:10.2196/31233)
Supplement: Multimedia Appendix 2 [file mhealth_v10i4e31233_app2.pdf]

Appendix 2: English Adolescent recruitment script page of the approved consent form

Open University of Catalonia
Baseline Survey
Mobile Application Pilot
Using Mobile Phone-Based Technologies To Provide On-Demand Adolescent Sexual Reproductive Health Information In A Resource Limited Setting: Kibra, Nairobi County

Introduction

Hello, my name is --------------------. **You** have been invited to take part in a research study. Before you decide whether to **participate**, you need to understand why the research is being done and what it would involve. Please take the time to read or to listen as I read the following information. You may talk to others about the study if you wish. Please ask me if there is anything that is not clear, or if you would like more information. When all your questions have been answered and you feel that you understand this study, you will be asked if you **will participate** in the study, and if you decide to **participate** in this research study, we will then ask you to visit the study site to give your **written assent/consent** to participate in the study.

Purpose of the Study and Study Requirements

What is the study about? This study is being carried out by **Paul Macharia of the Open University of Catalonia**. The study will pilot a mobile phone technology-based mode of accessing sexual reproductive health (SRH) information. The ultimate goal of this study is to improve the health and well-being of adolescents like ***you.***

Why have you been invited to take part? You have been invited to take part because **you** are an adolescent in this study site location.

What will happen if I take part?

1. **You** will be enrolled in a 3-month pilot of a mobile phone technology-based mode of accessing SRH information.

Risks

What are the risks of participating in the study? You may feel uneasy accessing Sexual Reproductive Health information in a mobile phone device.

Benefits

What are the benefits of participating in the study?
You will have an opportunity to be sensitized on adolescent SRH information and available services in this community, your participation will inform the design of a mobile phone technology-based system to bridge the SRH information gap which could enhance access to adolescent SRH information.

Are you willing to participate in the study?
