# Supplementary material for: An Unstructured Supplementary Service Data–Based mHealth App Providing On-Demand Sexual Reproductive Health Information for Adolescents in Kibra, Kenya: Randomized Controlled Trial
Source: JMIR Mhealth Uhealth. 2022 Apr 15;10(4):e31233. doi: 10.2196/31233 (PMC9055479; doi:10.2196/31233)
Supplement: Multimedia Appendix 3 [file mhealth_v10i4e31233_app3.pdf]

Appendix 3: Evaluation of Knowledge of SRH Information

Section 1: Sexual and reproductive health knowledge

a) How would you explain the female menstrual cycle?
   a. About once a month, females who have gone through puberty will experience menstrual bleeding.
   b. This happens because the lining of the uterus has prepared itself for a possible pregnancy by becoming thicker and richer in blood vessels.
   c. If pregnancy does not occur, this thickened lining is shed, accompanied by bleeding.
   d. Bleeding usually lasts for 3-8 days.
   e. No idea
   f. Other: Specify …………………………………………………………….

b) What are ways to avoid pregnancy?
   a. Condoms
   b. Contraceptive pills
   c. Intrauterine devices and implants
   d. Natural family planning
   e. No idea
   f. Other: Specify…………………………………………………………….

c) How do you think sexually transmitted infections (STIs) can be avoided?
   a. Abstinence
   b. Condom use
   c. Avoid substance use
   d. Sharing sex toys
   e. No idea
   f. Other: Specify …………………………………………………………….

d) What are symptoms of STIs?
   a. Genital/anal ulcer/sore
   b. Discharge from rectum
   c. Pain during defecation
   d. Burning pain during urination
   e. Urethral discharge
   f. Swelling in the groin
   g. Genital warts (penile or anal bumbs)
   h. Other: Specify…………………………………………………………….

Section 2: Adolescents who have "positive" attitudes toward SRH issues

1. Attitudes toward contraceptives
   a) Do condoms reduce sexual pleasure? **Yes [ ] No [ ]**
   b) Is carrying condoms difficult? **Yes [ ] No [ ]**
   c) Is using condoms a sign of mutual respect? **Yes [ ] No [ ]**
   d) Are condoms easy to obtain and use? **Yes [ ] No [ ]**
   e) Do unmarried adolescents not need to use condoms in all sexual encounters? **Yes [ ] No [ ]**
2. Gender-role stereotypes:
   a) Women who carry condoms are "easy" or prostitutes. **Yes [ ] No [ ]**
   b) Having sex with many women is a sign of manhood. **Yes [ ] No [ ]**
   c) "Real men" don't use condoms. **Yes [ ] No [ ]**

d) The female (sexual partner) is responsible for protection. **Yes [ ] No [ ]**

3. Attitudes toward abstinence:
   a) It is OK for adolescents to wait for marriage to have sex. **Yes [ ] No [ ]**
   b) My friends would laugh at me for refusing to have sex. **Yes [ ] No [ ]**
4. Perceived vulnerability:
   a) Pregnancy won‘t happen to me. **Yes [ ] No [ ]**
   b) STIs won‘t happen to me. **Yes [ ] No [ ]**
   c) Young people are healthy and don‘t need to worry about STIs. **Yes [ ] No [ ]**
   d) Women can get pregnant the first time they have sex. **Yes [ ] No [ ]**
