# Supplementary material for: An Unstructured Supplementary Service Data–Based mHealth App Providing On-Demand Sexual Reproductive Health Information for Adolescents in Kibra, Kenya: Randomized Controlled Trial
Source: JMIR Mhealth Uhealth. 2022 Apr 15;10(4):e31233. doi: 10.2196/31233 (PMC9055479; doi:10.2196/31233)
Supplement: Multimedia Appendix 4 [file mhealth_v10i4e31233_app4.pdf]

Appendix 4: Use and Perceptions of the Mobile Phone App

Have you ever used the mobile app for information on contraception, pregnancy, abortion, or sexually transmitted infections?	❖ **Yes**<br>❖ **No**	END
How many times have you used the mobile app for information in the last three (3) months?	Number of times …………..	END if 0 times
When you last used the mobile app, what information did you need? **(All that Apply)**	❖ Contraception<br>❖ STIs<br>❖ Sex<br>❖ Pregnancy<br>❖ Other……………………………	
Did you feel comfortable using the mobile app?	❖ **Yes**<br>❖ **No**	
Yes, Why?................................		
No, Why?................................		
Were the questions you had answered adequately?	❖ **Yes**<br>❖ **No**	
Yes, Why?................................		
No, Why?................................		
Did you feel that the information you received informed better decision making on SRH matters?	❖ **Yes**<br>❖ **No**	
Yes, Why?................................		
No, Why?................................		
If yes, what better decision making on SRH matters was informed by the information you accessed on the mobile app? **(All that Apply)**	❖ Condom use with partners<br>❖ Choice of Contraception<br>❖ Identifying an STI<br>❖ Abstinence<br>❖ Other: Specify…………	
Do you now feel knowledgeable about sexual reproductive health matters?	❖ **Yes**<br>❖ **No**	
Yes, Why?................................		
No, Why?................................		
If yes, what knowledge about sexual reproductive health matters have you gained? **(All that Apply)**	❖ Condom use with partners<br>❖ Choice of Contraception<br>❖ Identifying an STI<br>❖ Abstinence<br>❖ Other: Specify…………	
What do you think are the most important features of the mobile phone app on adolescent SRH? **(All that Apply)**	❖ Easy to use<br>❖ Quality information<br>❖ Confidential<br>❖ Immediate feedback<br>❖ Other: Specify ………….	
Was there enough confidentiality?	❖ **Yes**<br>❖ **No**	
Yes, Why?................................		
No, Why?................................		
